# Psychiatric Misdiagnosis in Frontotemporal Dementia

**DOI:** 10.1192/j.eurpsy.2022.2257

**Published:** 2022-09-01

**Authors:** A. Yay Pençe, İ. Ekmekçi Ertek, B. Coşar

**Affiliations:** Gazi University, Psychiatry Department, Ankara, Turkey

**Keywords:** misdiagnosis, FTD, differential diagnosis, frontotemporal dementia

## Abstract

**Introduction:**

Frontotemporal dementia(FTD) is the prevalent type of primary progressive dementia. Psychiatric symptoms can be seen in FTD. So it can imitate psychiatric disorders and be misdiagnosed. However, few studies have investigated the underlying cause of misdiagnosis.

**Objectives:**

The primary aim of this study was to identify the prior psychiatric diagnoses of patients before receiving a definitive diagnosis of FTD and the main reasons to cause diagnostic delay.

**Methods:**

We screened through the records of patients who were admitted to our psychiatry outpatient or inpatient clinic from January 1st, 2018 to June 30th, 2021. The patients with FTD were included in our study.

**Results:**

Our sample consisted of 13 patients with FTD(mean age= 54.77 ± 12.22, 7 females). Psychiatric misdiagnoses were depression(n=6), psychosis(n=5), bipolar affective disorder (n=5), conversion disorder(n=4), and malingering(n=1). As we looked at the first symptoms of the patients, it was revealed that 9 of 12 patients presented with depressive symptoms or at least experienced a short depressive period at the beginning of their behavioral changes. Interestingly, 8 of 12 patients had given a history of stressful life events just before their complaints emerged, which was thought the main misdirector for physicians. The average delays in diagnosis were 14.58(±16.93) months in the psychiatry clinic, 5.66(±11.02) months in the neurology clinic in our hospital.

**Conclusions:**

Our study suggests that the depressive episode preceding behavioral changes may be the prodromal stage for fully developed FTD. Moreover, the depressive episode and the history of stressful life events appear to mislead clinicians in diagnosing FTD.

**Disclosure:**

No significant relationships.

